# Roentgenological diagnosis of alimentary tract emergencies in the new born

**Published:** 2008-08

**Authors:** Kakarla Subbarao

## Introduction

With the rapid strides in the perfection of surgical techniques and with the advances in the pre and postoperative care of the patient, it is almost imperative to obtain an early and accurate pre-operative diagnosis of emergencies in the new born infant. It is a well documented fact that next to birth traumas, the commonest cause of death in the newborn is the complication of developmental anomalies. Roentgenological examination is of enormous importance in the early diagnosis of these emergencies of the alimentary tract as reliable objective evidence can be obtained at the time when the diagnosis is a matter of conjecture on the basis of clinical evidence alone. In the newborn there occur not infrequently, congenital anomalies, that if left untreated, result in death. Prompt recognition and adequate surgical correction of the anomaly will result in a gratifying low morality rate. Not only is the mortality in a given case directly related to the promptness of diagnosis and proper surgical treatment but also to the period of recovery and convalescence in the hospital which is uneventful and minimised to a considerable extent. It has been a common opinion among the medical profession that the newborn infant cannot withstand surgical procedures and that operations should be delayed on this ground whenever possible. But Ladd[7] has pointed out clearly that any infant during its first 48 hours of life presents a better surgical risk than it does a week or so later.

With the first breath of life air enters the pharynx, esophagus and then passes on into the stomach and intestines. As the baby goes on swallowing air, large amounts are visible in the small and large bowels within 12 hours after birth, depending upon the active crying of the baby. About 80% of the gas that is seen in the alimentary tract is due to swallowed atmospheric air. Experience tells us that, whenever the baby does not cry actively due to some reason or other, the amount of gas seen in the alimentary tract is slight in the first 12 hours. This presence of gas and its distribution in the small and large bowels serves as a contrast medium and helps in identifying the various anatomical segments of the alimentary tract and locate the site of obstruction when present.

## Esophageal Atresia

One of the gravest congenital anomalies is esophageal atresia with or without tracheoesophageal fistula. This anomaly is more common than is usually considered to be the case. There are several morphological types, the commonest being the one with atresia of the proximal end forming a blind pouch, the distal segment being in communication with the trachea or one of the main bronchi. According to Caffey[3] more than 70% communicate with the trachea or one of the primary bronchi through the inferior esophageal segment. The diagnosis is suggested in newborns and infants when there is excessive mucus at birth and prompt regurgitation of even small feedings, associated with attacks of cyanosis and respiratory difficulty. In cases where there is associated tracheoesophageal fistula, the scout film of the abdomen reveals gas in the stomach and intestines as inspired air passes through the patent inferior esophageal segment. In the less common types without tracheoesophageal fistula the abdomen is scaphoid and deviod of gaseous content. In either type of atresia there is present the complication of aspiration of ingested material as well as mucus into the lungs causing atelectasis and pneumonia, especially of the right upper lobe.

**Figure 1:**
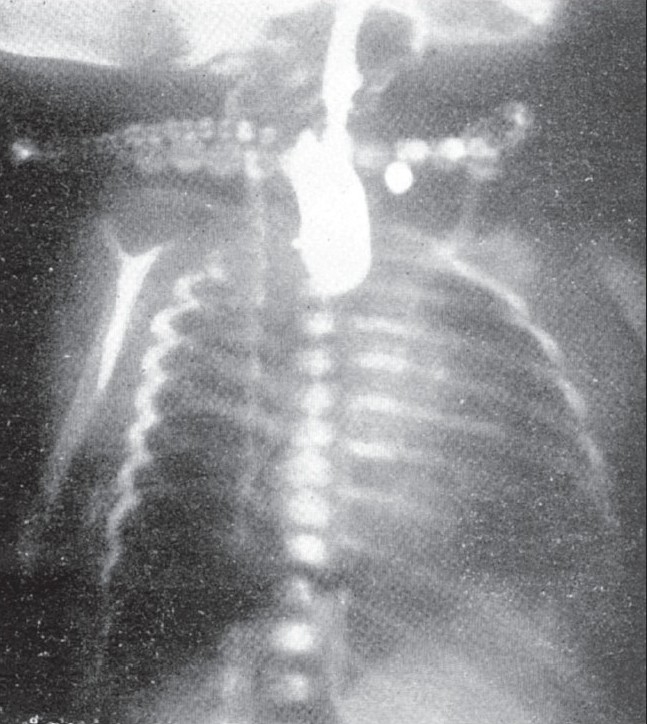
Esophageal atresia. The blind pouch filled with Lipiodol is quite characteristic of this condition

The roentgenoligical diagnosis is confirmed by passing a soft rubber catheter into the esophagus and finding an obstruction at about the level of the second or third dorsal vertebrae. This procedure as well as the injection of one or two c.c. of lipiodol through the catheter to outline the upper esophageal segment and its blind pouch should be performed under fluoroscopic control. Such an examination with the help of a spot film demonstrates the level of the upper esophageal pouch as seen in Figure 1, and determines the presence or absence of a fistula connecting the upper esophagus and trachea. This technique of radiographic examination is much safer than administration of barium by mouth. With the latter procedure, a portion of regurgitated barium is almost invariably aspirated into the lungs which results in a severe and often fatal pneumonia.

## Forked or Annular Pancreas

**Figure 2:**
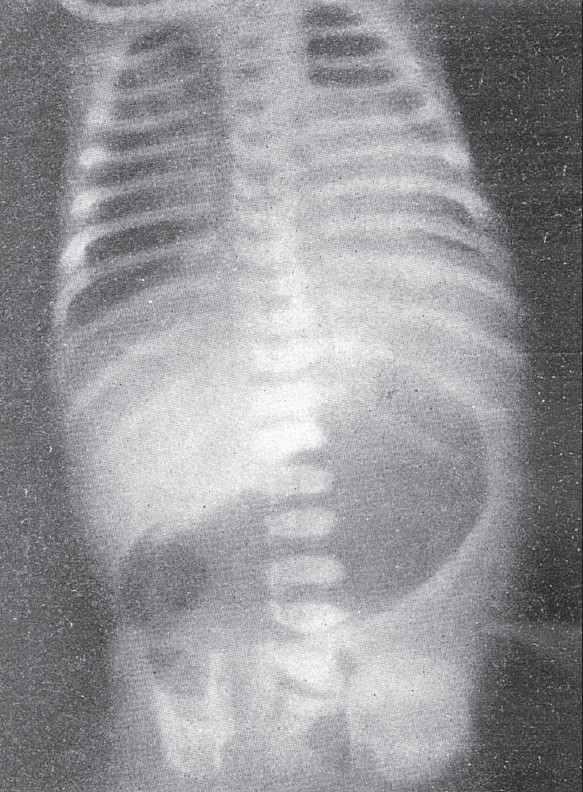
Forked pancreas. Note the distended stomach and duodenal cap with very little gas distal to the obstruction

This is a congenital anomaly of indeterminate origin and as a rule the upper descending portion of the duodenum is involved but any portion may be encircled. The circle may not be complete always. It is a rather rare extrinsic cause of congenital duodenal obstruction. Slight degrees of obstruction go undetected throughout life. One of Poppel's[11] cases was associated with carcinoma of the head of pancreas in a 65-year-old man. When the obstruction is complete the child is seen early in life with persistent vomiting. A scout film of the abdomen taken at that time reveals a distended stomach and duodenal cap. The markedly distended stomach and proximal duodenum with the presence of minimal amounts of gas in the distal portion of the intestines as shown in Figure 2, are rather characteristic of annular or forked pancreas causing incomplete obstruction of the duodenum. However it is very often impossible to differentiate this from obstruction caused by other rarer anomalies such as peritoneal or periduodenal bands. In cases where it is hard to postulate a diagnosis of duodenal obstruction on scout films of the abdomen alone, it is advisable to administer contrast material by mouth and thus study the alimentary tract.

## Intestinal Atresia or Stenosis

Acute intestinal obstruction in the new born, is sometimes produced by atresia or stenosis of a segment of the small bowel. This is the commonest cause of intrinsic intestinal obstruction resulting from failure of complete re-canalization of the epithelial tube in embryonic life. Atresia results from an imperforated intestinal diaphragm or a solid occluding epithelial plug, whereas stenosis results when small channels exist through these diaphragms or plugs. Attention is directed to these patients because of persisting vomiting of bile-stained material, which starts soon after birth. The site of obstruction usually determines the abdominal distention.

**Figure 3 (A, B):**
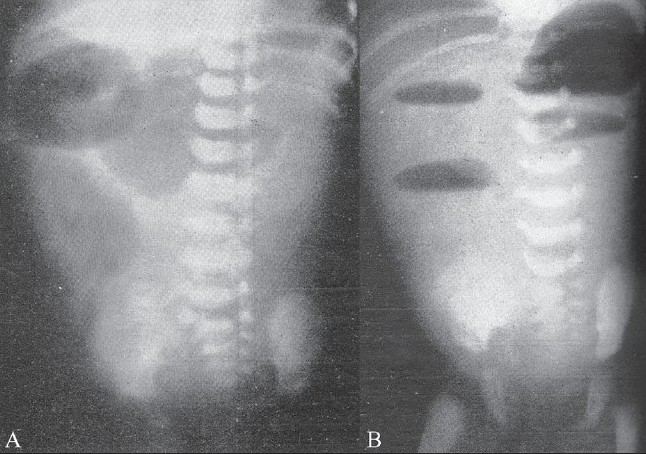
Supine (A) and erect (B) radiographs show a markedly dilated stomach and duodenal loop proximally in the supine position with air-fluid levels in the upright position

**Figure 4:**
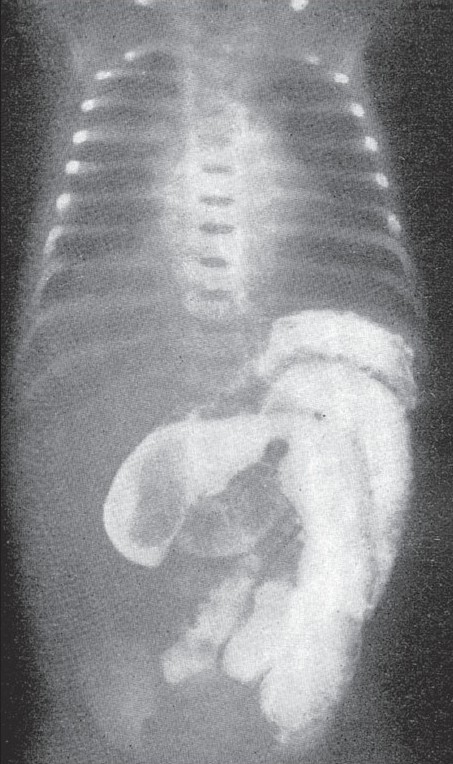
Atresia of the ileum. Note the barium distended small bowel with conspicuous absence of gas in the terminal ileum and large bowel

Roentgenological examination should be limited to scout films of the abdomen in various views wherever it is possible. From the scout films alone, the point of obstruction could be located as no gas is found beyond the stenotic segment. In case of complete intestinal obstruction, barium should never be administered by mouth. As has been mentioned before, the child might aspirate the barium into the lungs during the act of vomiting or the barium might form a concretion in the bowel, which becomes a hazard to the patient post-operatively after an anastomosis circumventing the atresia has been made. When the atresia or stenosis is in the terminal loops of the ileum and when there is enormous gaseous distension proximally, at times it is difficult to distinguish it from a large bowel distension. As a rule, in all doubtful cases to make out whether it is small or large bowel obstruction, it is always safer to do a barium enema. This helps in ruling out a large bowel obstruction Figure 3 represents a case of complete atresia of a segment of small intestine, extending from the distal duodenum to the proximal jejunum, involving 15 cm of small bowel. In all these cases of obstruction, either an erect or a recumbent lateral film of the abdomen shows the air-fluid levels characteristic of intestinal obstruction. A barium enema also helps in locating the cecum so that any associated nonrotations of the gut can be made out before laparoscopy. Figure 4 represents a case of atresia of a segment of the ileum with volvulus of the involved portion. In this case, barium was administered by mistake, but was promptly aspirated before the operation. The distended proximal loops of bowel as outlined by the contrast material and the absence of gas distal to the obstruction are characteristic of mechanical obstruction, which in the newborn should suggest the diagnosis of some sort of intestinal anomaly.

## Meconium Ileus

This pathological entity owes its recognition mainly to Farber[5] and Neuhauser[9]. This is caused by pancreatic insufficiency, the common cause of which is congenital stenosis of the pancreatic ductal system. In these cases, the quality of pancreatic enzyme (trypsin) reaching the intestines is altered and the meconium is thicker and sticky. The fetal intestine is unable to propel this and in a certain number of cases, the lumen becomes occluded, often in the terminal third of the ileum. There is early onset of vomiting after birth with progressive abdominal distension.

The roentgenological findings are basically similar to any type of small bowel obstruction. In cases of complete obstruction, the scout films reveal pronounced distension of the small bowel with air-fluid levels demonstrable in the erect or lateral recumbent postures. However, in some cases, a small amount of gas is forced into the tenacious and mucilaginous meconium and is seen as small bubbles of gas scattered in the small bowel. Neuhauser[9] has pointed out that a definite diagnosis can be arrived at in about 40% of cases on the basis of scout films of the abdomen in all types of cases of meconium ileus. If the fundamental etiological process involves the bronchioles also, as has been described in a number of cases, patchy atelectasis and obstructive emphysema are seen in the film of the chest taken in the earlier stages. In the later stages of the disease, with superimposed infection, the chest films might reveal isolated areas of atelectasis, patchy bronchopneumonia or even brochiectasis, depending upon the degree of involvement.

**Figure 5:**
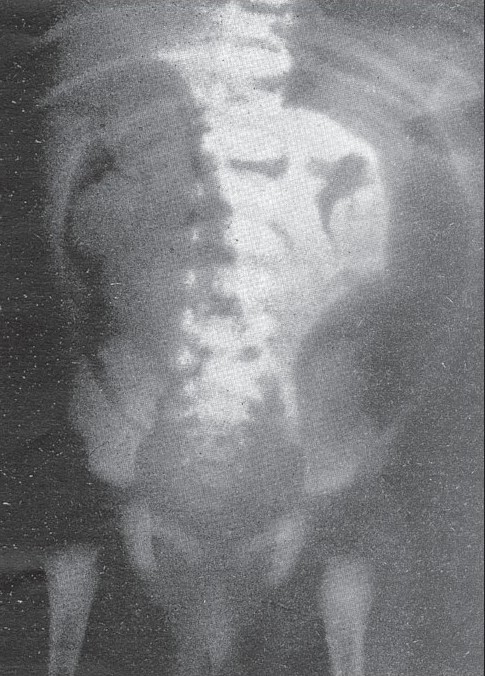
Meconium ileus with volvulus. The air bubbles in the meconium of the ileum, which is twisted, are quite diagnostic. The colon is outlined by gas induced by a soap and water enema

The rarer complication of volvulus of the segment of bowel occluded with meconium should be borne in mind when it becomes a real surgical emergency. Figure 5 represents a case of meconium ileus in a premature baby. The characteristic meconium with bubbles of air led us to an erroneous diagnosis of volvulus of the sigmoid. The large bowel is outlined peripherally by gas which was induced during an ordinary soapwater enema. The calibre of he non-functioning large bowel is significantly small.

## Meconium Peritonitis

**Figure 6 (A, B):**
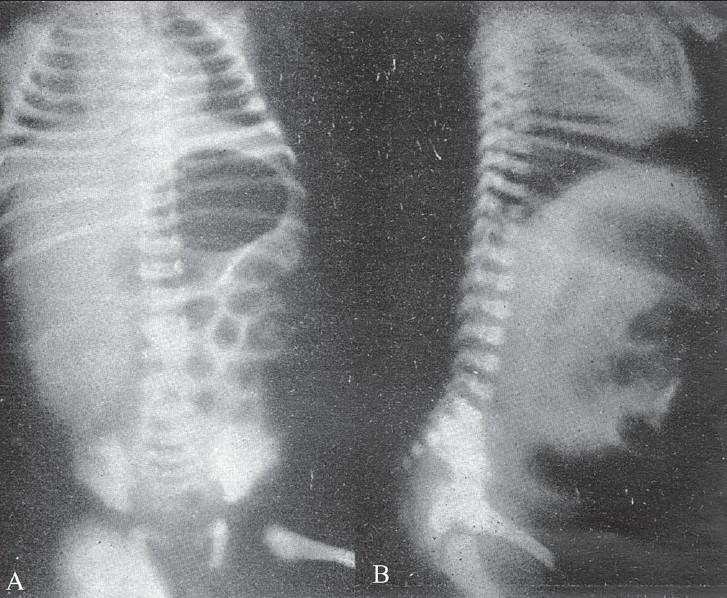
AP (A) and lateral (B) views show meconium peritonitis. Note the plaquey calcifications

This disorder is a nonbacterial chemical peritonitis, which results from the extrusion of meconium through the intestinal wall into the peritoneal cavity. Whether this process goes on in late fetal life or immediately after birth is not definitely know. Variable amounts of lime are usually deposited in the extruded meconium in the form of incrustations on the clusters of cornified epithelial cells. When the calcified masses are large enough they can be visualised on the scout films of the abdomen. These calcifications are usually circular or curvilinear, irregularly distributed in the abdomen. These calcifications associated with small intestinal obstruction, are fairly characteristic of meconium peritonitis. Figure 6 is an example of a case of meconium peritonitis with calcification in the right side of the abdomen.

## Atresia of the Rectum Associated with Imperforated Anus

Imperforate anus is one of the most frequent emergencies in the newborn and is promptly recognised without an X-Ray examination. The complexity is not appreciated however, since this anomaly is commonly associated with atresia of the rectum and some type of a fistula; rectovaginal, rectoperineal, rectourethral or rectovesical. In some cases, the rectum and the rest of the colon are normal except for a cutaneous septum in the region of the anus. An infant with this type of imperforate anus will rapidly develop marked gaseous distension of the intestines within a few hours of birth. It is of prime importance to determine how low the rectal pouch is in the pelvis to help in determining the type of operation that is going to be employed.

**Figure 7:**
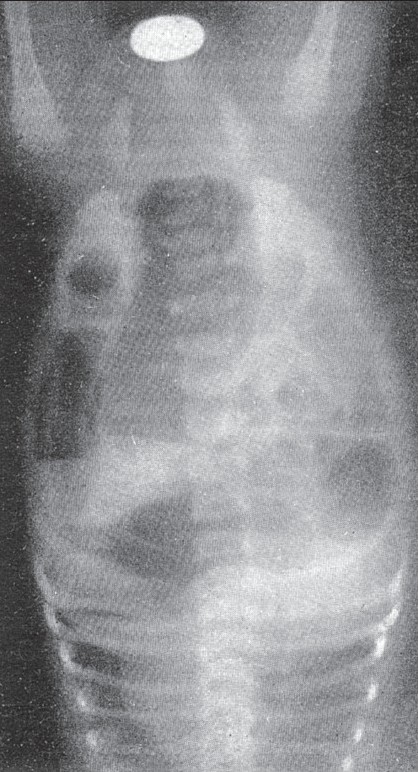
Imperforate anus. Wangensteen's technique in the inverted position with a metallic marker. Gas outlines the rectum

Using Wangensteen's technique of inverting the baby and placing a metallic marker on the perineal dimple, it is possible to determine how near the pouch is to the perineum by taking a lateral view of the pelvis (Figure 7). This method of roentgenological examination is only valid after 14-18 hours of life. Otherwise gas may not have reached the rectal pouch. The fistulous tracts if present can be visualized after injection of lipiodol and taking anterio-posterior and lateral views of the pelvis. The tracts running to the genito-urinary system should be easily traced.

## Diaphragmatic Anomalies

**Figure 8 (A, B):**
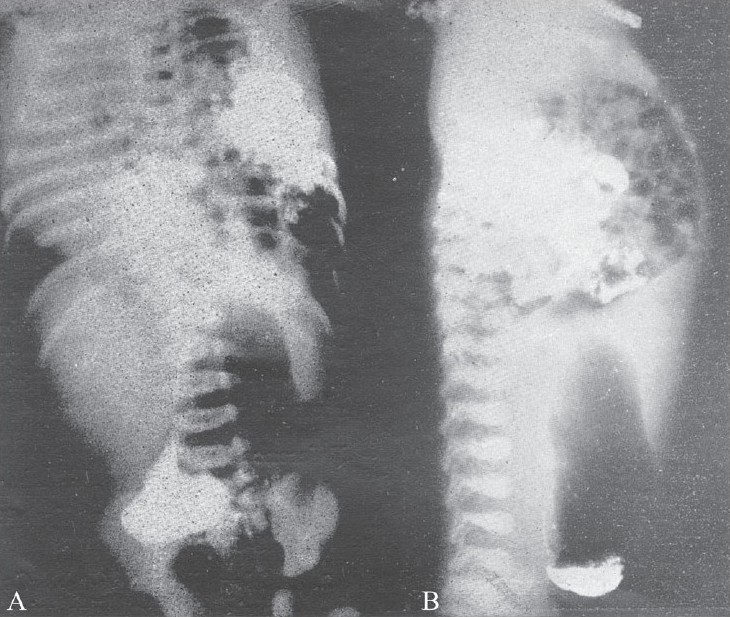
Diaphragmatic hernia. AP (A) and lateral (B) radiographs show shift of the heart and mediastinum with Lipiodol-outlined bowel

Partial or complete absence of a hemidiaphragm is not so common as is thought to be. Unless it is recognized early and treated radically, the morality rate approaches 80-90%. Of all varieties of anomalies of the diaphragm, the commonest is the defect in the pleuroperitoneal membrane region, especially on the left side. This results in massive herniation of the abdominal contents into the thoracic cavity. The newborn presents with characteristics attacks of cyanosis associated with dyspnea particularly during or shortly after feedings. Roentgenological examination of the chest and abdomen presents the true picture of the herniation. The chest reveals the heart and mediastinum to be pushed to the opposite side of the hernia. The presence of gas distended gastro-intestinal tract in the thoracic cavity cannot be mistaken for any other condition. One may administer lipidiol by mouth for identification and location of various portions of the gastrointestinal tract, and thus confirm the diagnosis as well Figure 8 represents a case, brought to the roentgenology department with a possible diagnosis of dextrocardia associated with some other congenital heart disease. Examination of the chest revealed it to be herniation through the left hemidiaphragm. There is marked shift of the heart and mediastinum to the right side causing cardiac and respiratory embarrassment, which explains the clinical impression.

## Omphalocele

**Figure 9:**
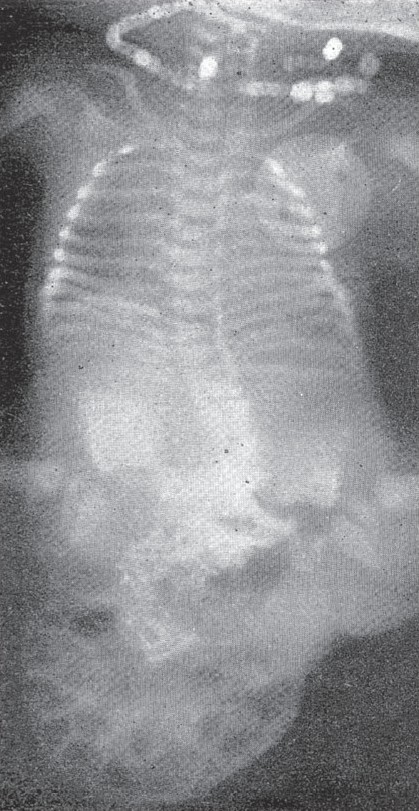
Omphalocele. Enormous umbilical hernia containing all the bowel. Incidentally noted teeth in a congenital sacro-coccygeal teratoma

This anomaly of developmental origin is such a striking abnormality in the newborn that it usually receives prompt attention by the clinician. This condition consists of herniation of the abdominal contents into an abnormal sac-like umbilical cord, which consists of a translucent membrane composed of peritoneum and amniotic membrane. In rare cases, the infant may be born with an omphalocele in which the sac is absent. Roentgenological examination is imperative whenever there is clinical evidence of intestinal obstruction or strangulation. Anteroposterior and lateral views of the abdomen reveal the typical obstruction pattern of the bowel as previously described. In addition, the gas distended loops of bowel can be traced lying outside the anterior abdominal wall. However, occasionally associated abnormalities such as sacrococcygeal dermoid can also be detected as in one of our cases which is shown in Figure 9.

## Miscellaneous

Malrotations of the gastrointestinal tract and faulty mesenteric attachment may rarely lead to volvulus of the mid or hind-gut. In these cases, scout films of the abdomen suggest a picture of intestinal obstruction but do not help usually in making an etiological diagnosis. In cases of non-rotation of the hind-gut, a barium enema of the colon reveals the abnormal position of the cecum, which will be situated on the left side of the abdomen.

The much rarer anomalies such as pericardiodiaphragmatic herniation and reduplications of the alimentary tract with complications of obstruction and strangulation of the intestines deserve mention and roentgenological examination with contrast media plays a very important role in the postulation of a diagnosis preoperatively.

## Summary

An attempt has been made to stress the importance of roentgenological examination in establishing definite preobservation diagnosis in some of the alimentary tract emergencies in the newborn. A brief account of the roentgenological criteria has been given with illustrations of surgically proved cases whereever possible. A short list of pertinent references is appended.
